# Supplementary material for: Empirical study on visual attention characteristics of basketball players of different levels during free-throw shooting
Source: PeerJ. 2023 Dec 11;11:e16607. doi: 10.7717/peerj.16607 (PMC10720415; doi:10.7717/peerj.16607)
Supplement: Supplemental Information 1 [file peerj-11-16607-s001.docx]

| **Table 1 Comparison of average number of fixations of participants with different levels in each area of interest(times)** | | | | | | | | | | | |
| --- | --- | --- | --- | --- | --- | --- | --- | --- | --- | --- | --- |
|  | **Expert group** | | | **General group** | | **Novice group** | | | |  | |
| **AOI** | ***M*** | ***SD*** | ***M*** | | ***SD*** | | ***M*** | ***SD*** | ***F*** | | ***P value*** |
| Front | 1.30 | 0.47 | 1.50 | | 0.51 | | 1.80 | 0.52 | 5.014 | | 0.010^*^ |
| Top | 1.20 | 0.41 | 1.35 | | 0.49 | | 1.45 | 0.51 | 1.421 | | 0.250 |
| Bottom | 0.95 | 0.22 | 1.25 | | 0.44 | | 1.35 | 0.49 | 5.341 | | 0.007^*^ |
| Top left | 1.05 | 0.22 | 1.20 | | 0.41 | | 1.25 | 0.44 | 1.563 | | 0.218 |
| Bottom left | 0.90 | 0.30 | 0.95 | | 0.22 | | 1.00 | 0.00 | 1.036 | | 0.361 |
| Top right | 0.05 | 0.24 | 0.70 | | 0.47 | | 0.95 | 0.22 | 40.336 | | 0.000^* *^ |
| Bottom right | 0.05 | 0.22 | 0.65 | | 0.49 | | 0.90 | 0.31 | 29.801 | | 0.000^* *^ |
| **Notes**.  **P*＜0.05  ***P*＜0.01 |  |  |  | |  | |  |  |  | |  |

| Table 2.Comparison of saccadic amplitude among different exercise level participants.( °) | | | | |
| --- | --- | --- | --- | --- |
| **Group** | ***M*** | ***SD*** | *F* | *P* ***value*** |
| expert | 3.53 | 0.30 | 4.660 | 0.13^*^ |
| general | 3.54 | 0.14 |  |  |
| novice | 3.72 | 0.19 |  |  |
| **Notes**. |  |  |  |  |
| ^*^*P*＜0.05 |  |  |  |  |
| ^**^*P*＜0.01 |  |  |  |  |

| T**able 3 Comparison of fixation duration** **participants with different levels（MS）** | | | | | | | | | | |
| --- | --- | --- | --- | --- | --- | --- | --- | --- | --- | --- |
|  | **expert** | | **general** | | | | **novice** | |  | |
| **AOI** | ***M*** | ***SD*** | | ***M*** | ***SD*** | ***M*** | | ***SD*** | ***F*** | ***P value*** |
| Front | 628.50 | 92.92 | | 591.00 | 63.24 | 476.00 | | 56.33 | 23.970 | 0.000** |
| Top | 563.00 | 116.71 | | 542.60 | 53.87 | 365.00 | | 50.32 | 37.345 | 0.000** |
| Bottom | 319.00 | 84.16 | | 367.50 | 37.96 | 368.00 | | 57.36 | 4.023 | 0.023* |
| Top left | 336.75 | 32.05 | | 364.30 | 55.19 | 360.50 | | 59.60 | 1.754 | 0.182 |
| Bottom left | 225.25 | 85.43 | | 230.00 | 64.81 | 250.00 | | 49.84 | 0.740 | 0.482 |
| Top right | 15.00 | 67.08 | | 148.50 | 100.75 | 208.50 | | 55.66 | 27.665 | 0.000** |
| Bottom right | 10.00 | 110.18 | | 141.50 | 110.18 | 200.00 | | 76.09 | 25.476 | 0.000** |
| **Notes** |  |  | |  |  |  | |  |  |  |
| *^*^P*＜0.05 |  |  | |  |  |  | |  |  |  |
| *^**^P*＜0.01 |  |  | |  |  |  | |  |  |  |

| Table 4 Comparison of pupil dilation of participants with different levels（MM） | | | | |
| --- | --- | --- | --- | --- |
| **Group** | ***M*** | ***SD*** | ***F*** | ***P value*** |
| expert | 1 384.95 | 90.03 | 8.503 | 0.001^**^ |
| general | 1 333.60 | 57.59 |  |  |
| novice | 1 300.00 | 38.65 |  |  |
| **Notes** |  |  |  |  |
| *^*^P*＜0.05 |  |  |  |  |
| *^**^P*＜0.01 |  |  |  |  |
